# Evaluating the utility of *ZNF331* promoter methylation as a prognostic and predictive marker in stage III colon cancer: results from CALGB 89803 (Alliance)

**DOI:** 10.1080/15592294.2024.2349980

**Published:** 2024-05-08

**Authors:** Elizabeth S. Nakasone, Tyler J. Zemla, Ming Yu, She Yu Lin, Fang-Shu Ou, Kelly Carter, Federico Innocenti, Leonard Saltz, William M. Grady, Stacey A. Cohen

**Affiliations:** a Division of Oncology, University of Washington, Seattle, WA, USA; b Translational Science and Therapeutics Division, Fred Hutchinson Cancer Center, Seattle, WA, USA; c Alliance Statistics and Data Management Center, Mayo Clinic, Rochester, MN, USA; d School of Life Sciences, Nantong University, Nantong, P.R. China; e Division of Pharmacotherapy and Experimental Therapeutics, University of North Carolina at Chapel Hill, Chapel Hill, NC, USA; f Department of Gastrointestinal Oncology, Memorial Sloan Kettering Cancer Center, New York, NY, USA; g Division of Gastroenterology, University of Washington, Seattle, WA, USA

**Keywords:** CALGB 89803 (Alliance), epigenetic factors, DNA methylation, ZNF331, biomarker, stage III colon cancer

## Abstract

While epigenomic alterations are common in colorectal cancers (CRC), few epigenomic biomarkers that risk-stratify patients have been identified. We thus sought to determine the potential of *ZNF331* promoter hypermethylation (m*ZNF331*) as a prognostic and predictive marker in colon cancer. We examined the association of m*ZNF331* with clinicopathologic features, relapse, survival, and treatment efficacy in patients with stage III colon cancer treated within a randomized adjuvant chemotherapy trial (CALGB/Alliance89803). Residual tumour tissue was available for genomic DNA extraction and methylation analysis for 385 patients. *ZNF331* promoter methylation status was determined by bisulphite conversion and fluorescence-based real-time polymerase chain reaction. Kaplan-Meier estimator and Cox proportional hazard models were used to assess the prognostic and predictive role of m*ZNF331* in this well-annotated dataset, adjusting for clinicopathologic features and standard molecular markers. m*ZNF331* was observed in 267/385 (69.4%) evaluable cases. Histopathologic features were largely similar between patients with m*ZNF331* compared to unmethylated *ZNF331* (unm*ZNFF31*). There was no significant difference in disease-free or overall survival between patients with m*ZNF331* versus unm*ZNF331* colon cancers, even when adjusting for clinicopathologic features and molecular marker status. Similarly, there was no difference in disease-free or overall survival across treatment arms when stratified by *ZNF331* methylation status. While *ZNF331* promoter hypermethylation is frequently observed in CRC, our current study of a small subset of patients with stage III colon cancer suggests limited applicability as a prognostic marker. Larger studies may provide more insight and clarity into the applicability of m*ZNF331* as a prognostic and predictive marker.

## Introduction

Colorectal cancer (CRC) is a group of heterogeneous diseases that arise from the gradual accumulation of genetic and epigenetic alterations [1]. A major driver of carcinogenesis is the development of specific molecular phenotypes that give rise to genomic instability, most commonly through chromosomal instability (CIN) or microsatellite instability (MSI). CIN results in chromosomal gains, losses, and translocations. MSI arises from disruptions in DNA mismatch repair (MMR) mechanisms, leading to the progressive accumulation of gain- and loss-of-function mutations [2,3].

In addition to genomic instability, aberrant epigenomic alterations are also frequently observed in CRC, typically through covalent histone modification and DNA methylation [4–6], affecting transcription without altering the underlying genomic sequence. These transmissible alterations have been increasingly identified as contributors to both CRC carcinogenesis and disease progression through activation of proto-oncogenes in hypomethylated states and transcriptional silencing of tumour suppressor genes in hypermethylated states [7]. Epigenetic modification can affect a single gene or be genome wide. The hallmark example of the former in CRC is hypermethylation of the *MLH1* promoter, which leads to loss of MLH1 expression and tumour microsatellite instability [8,9]. On a more global scale, a specific and reproducible pattern of genome-wide hypermethylation can occur in CRC termed the CpG island methylator phenotype (CIMP) [10].

Since the initial discovery of the gene mutations and aberrant DNA methylation in cancer, there has been great interest in identifying specific DNA-based biomarkers that are not only prognostic but may also predict individualized treatment responses [11]. To date, epigenomic alterations have been demonstrated to be most valuable as diagnostic markers of CRC, as aberrant patterns of promoter methylation appear to occur early in carcinogenesis and are highly sensitive and specific for CRC [12].

With regard to methylated DNA biomarkers, CIMP remains the most recognized predictive and prognostic epigenomic biomarker for patients with CRC. Indeed, we and others have previously shown that CIMP+ tumours are associated with specific clinicopathologic features including early stage, location in the proximal colon, poor or mucinous histopathology, and *KRAS* and *BRAF*^V600E^ mutations [13–17]. As noted above, there is overlap between CIMP and methylated *MLH1*-mediated MSI. However, implementation of CIMP as a reliable predictive biomarker of CRC in clinical care has been limited, due in part to variability in methods of assessment, leading to heterogeneity in treatment response data.

A putative biomarker that has garnered attention is promoter hypermethylation of *ZNF331* (m*ZNFF331*). m*ZNF331* has been observed in a variety of malignancies, including gastric, pancreatic, bile duct, and colorectal cancers [18,19]. In CRC, m*ZNF331* is frequently observed in both cell lines and primary human tumour specimens, with little to no promoter methylation detected in matched normal control specimens [19,20]. Furthermore, m*ZNF331* is associated with reduced overall and disease-free survival [20,21]. Limited preclinical data suggest that loss of *ZNF331* expression reduces cancer cell proliferation *in vitro* and *in vivo* and enhances invasive capacity *in vitro*. Overexpression of ZNF331 can reverse these acquired properties [18,20,22].

We therefore conducted this analysis to evaluate the utility of *ZNF331* promoter hypermethylation as a prognostic and/or predictive marker in stage III colon cancer patients enrolled in a large, well-annotated randomized phase III clinical trial.

## Materials and methods

### Study population

As previously described [23], patients evaluated in this study were enrolled in the Cancer and Leukaemia Group B (CALGB) 89803 (NCT0003835) adjuvant phase III trial comparing 5-fluorouracil/leucovorin (FU/LV) alone or in combination with irinotecan (IFL). Primary endpoints of the study were overall survival (OS), measured from the time of study entry to death due to any cause, and disease-free survival (DFS), defined as the time from study entry until tumour recurrence or death from any cause. Secondary aims were to evaluate the relationship between tumour-associated risk factors and treatment outcomes. A total of 1264 patients were enrolled and randomly assigned to the FU/LV or IFL treatment arm using a fixed block strategy between April 1999 and May 2001. Follow-up was completed on 9 November 2009.

### Ethics statement

CALGB is now part of the Alliance for Clinical Trials in Oncology (Alliance). The CALGB/Alliance Statistics and Data Management Center maintains the clinical and laboratory database. All patients provided written informed consent prior to participation in this trial, and this protocol was approved by the institutional review board of each participating centre. All patients enrolled in CALGB/Alliance 89803 also provided written informed consent for additional molecular testing for correlative biomarker studies. This biomarker study was approved by the Fred Hutchinson Cancer Center institutional review board (IR 1989). The analysis herein presented represents a correlative biomarker analysis on a subset of patients from whom residual tumour tissue was available for genomic DNA (gDNA) extraction and methylation analysis.

### Treatment

As previously published [23], patients randomized to the FU/LV arm received the Roswell Park regimen consisting of LV 500 mg/m^2^ by intravenous (IV) injection over 2 hours, with an IV bolus injection of FU 500 mg/m^2^ at 1 hour after initiation of LV. Treatments were administered weekly for 6 consecutive weeks, followed by a 2-week rest period, for a total of four cycles over 32 weeks. Patients randomized to the IFL arm received irinotecan 125 mg/m^2^ over 90 minutes followed by an IV bolus injection of LV 20 mg/m^2^ and then an IV bolus injection FU 500 mg/m^2^. Treatments were administered for four consecutive weeks followed by a 2-week rest period for five cycles or a total of 30 weeks.

### DNA extraction from tumor specimens

Tumour molecular analyses were performed blinded to patient and outcome data. All assays were performed in a non-Clinical Laboratory Improvement Amendment (CLIA)-approved research laboratory at the Fred Hutchinson Cancer Center (William M. Grady, Principal Investigator). Tissue sections 10 μm in thickness were cut from formalin-fixed and paraffin-embedded (FFPE) blocks. A haematoxylin and eosin-stained slide was prepared for histopathologic review and was used to guide microdissection of adjacent tissue sections. Microdissection was performed using sterile razor blades to obtain specimens containing > 70% tumour content. The QIAamp DNA FFPE Tissue Kit (Qiagen) was used to extract genomic DNA from FFPE tissues according to manufacturer’s instructions. Samples were then eluted into 50–100 μL, dependent upon tissue size and kit protocol recommendations. Quant-iT PicoGreen DNA assay kit (Life Technologies) was used to quantify genomic DNA before bisulphite conversion.

### Sodium bisulfite conversion and recovery of genomic DNA

DNA samples (1 μg) were bisulphite converted using the EZ DNA Methylation Kit (ZYMO Research, Irvine, CA, USA) as per manufacturer protocol, with a final eluted volume of 20 μL. Converted DNA was diluted 1:10 for methylation assays.

### ZNF331 methylation analysis

After bisulphite conversion, gDNA was analysed by a quantitative MethyLight method for detecting methylated *ZNF331* (NM_018555.6, hg19), a fluorescence-based, quantitative methylation-specific polymerase chain reaction (PCR), as described in a previous study [19] with the following modifications. The *ZNF331* Methylight was performed using a CFX96^TM^ thermal cycler (Bio-Rad, Hercules, CA) in a 96-well format with a primer and probe set designed to detect *ZNF331* methylation. A 25-μL reaction mixture (20 ng of bisulphite-converted gDNA, 10 pmol of each primer, and 12.5 μL of iTaq™ Universal Probes Supermix (Bio-Rad, Hercules, CA) and was cycled under the following conditions: 95°C for 30 seconds followed by 45 cycles of 95°C for 15 seconds and 60°C for 1 minute. Each reaction was run in duplicate. No template, positive (100% methylated), or negative (100% unmethylated) DNA controls were run with each reaction. These results were scored as percent of methylated reference values. Data were analysed using the Bio-Rad CFX manager software version 3.1 (BioRad, Hercules, CA) and quantification cycle (Cq) was determined with the Single Threshold method. To assess the specificity of MethyLight PCR to only detect methylated alleles, an initial experiment was conducted using 100% unmethylated bisulphite-converted EpiTect Unmethyl control DNA (QIAGEN Cat #59665). We didn’t detect amplification signal in those samples. Non-template-control (NTC) wells gave no amplification signal. We routinely evaluate each MethyLight reaction by including positive control wells containing 4,500 pg of 100% methylated EpiTect Methyl control DNA, negative control wells containing 4,500 pg of 100% unmethylated EpiTect Unmethyl control DNA, and NTC wells. We performed a dilution experiment to determine the sensitivity and quantitative accuracy of the *ZNF331* Methylight assay. To evaluate the primer efficiency, a five-point standard curve was determined using serial dilutions of the 100% methylated EpiTect Methyl control DNA (QIAGEN Cat #59655) spanning a range of 45,000 pg, 4,500 pg, 450 pg, 45 pg, and 4.5 pg per reaction. We also optimized the primer concentration and annealing temperatures to achieve the optimal assay performance. Intra-assay variation in concentration measured by percent coefficient of variation was less than 15%. A methylation-independent *ALU-C4* control reaction was used to normalize input DNA amounts as previously described [24]. The percent methylated reference was set at 4% per literature standards [25] with m*ZNF331* defined as ≥ 4% methylation.

The primers and probes for *ZNF331* MethyLight reaction were designed using the Primer Express Software 3.0 (Life Technologies), as previously described [19]. The primer and probe sequences for methylated *ZNF331* and *ALU-C4* are listed in Supplemental Table S1.

### Analyses of microsatellite instability, CIMP, and KRAS, BRAF, and TP53 mutations

Tumour molecular analysis was performed on DNA extracted from archival FFPE tissue blocks as previously described. *KRAS* mutations (exons 2, 3, and 4) [26–28] and *BRAF*^V600E^ mutations (exon 15) [27,28] were assessed using dynamic decoupling sequencing on nested PCR amplicons at the Laboratory of Molecular Oncology (Hellenic Foundation for Cancer Research, Aristotle University of Thessaloniki School of Medicine, Thessaloniki, Greece). *TP53* mutations (exons 5–8) [29,30] were assessed by direct sequencing or sequencing by hybridization at the University of California, San Francisco Genomics Core Facility (University of California, San Francisco, San Francisco, CA, USA) as previously described. Microsatellite instability was determined by immunohistochemical staining for MMR protein expression and/or by PCR using microsatellite markers defined by the 1998 National Cancer Institute Workshop on Microsatellite Instability [31,32] at the Brigham and Women’s Hospital in Boston, MA, USA [32,33]. CIMP [17] was determined by assessing methylation of promoter CpG islands was performed on a previously validated 5-gene marker panel (*RUNX3*, *CACNA1G*, *IGF2*, and *NEUROG1*, *MLH1*) [34] by MethyLight assay. A percentage of methylated reference of ≥ 4% was used to define the threshold of methylation [25]. CIMP+ status was defined as methylation of ≥ 3 of 5 methylated loci.

### Statistical methods

Baseline clinical patient characteristics were compared between patients with methylated and unmethylated *ZNF331* (m*ZNF331* and un*ZNF331*, respectively). Continuous variables were presented as medians with interquartile percentiles (IQR), whereas categorical variables were expressed as counts and percentages. Univariate comparisons were performed using the Wilcoxon rank-sum test [35] for continuous variables and Pearson Chi-squared test [36] for categorical variables. Follow-up was limited to 8 years, and so patients who did not experience an event by this time point were right censored. The distribution of time-to-event endpoints (overall survival [OS], disease-free survival [DFS]) were estimated by Kaplan – Meier curves [37]. OS and DFS between different biomarker categories were tested using log-rank test [37]. Multivariable Cox regression [38] was used to assess the prognostic associations of *ZNF331* status with OS and DFS, adjusting for other key clinical-pathological factors (age, gender, performance status, tumour site, T-stage, number of positive nodes, and histologic grade) and treatment arms. CIMP status was entered in the model as an additional adjusted variable. No imputation was done for covariates with missing values. The prognostic effect of *ZNF331* status was further examined within each key biomarker status (*BRAF*, *TP53*, and *KRAS* mutational status, MMR status and CIMP) by incorporating an interaction term between the *ZNF331* methylation status and key biomarker in the Cox model. The predictive effect of *ZNF331* methylation was examined by including methylation status, treatment arm, and the interaction between the two in the Cox model while adjusting for key clinicopathologic factors mentioned above. To further examine the predictive effect of *ZNF331* status within specific key biomarker status, Cox models with *ZNF331* status, treatment arm, key biomarker, and the 2-way/3-way interactions between the three were used while adjusting for key clinical-pathological factors mentioned above. The treatment effect (IFL versus FU/LV) in each methylation and biomarker status was estimated using the point estimates from the Cox model. The *p*-value of the interaction term was calculated based on the likelihood ratio test. Analyses were done with SAS (version 9.4; SAS Institute, Cary, NC, USA). Two-sided *p* values of less than 0.05 were considered significant and were not adjusted for multiple comparisons. Data collection and statistical analyses were conducted by the Alliance Statistics and Data Management Center. Data quality was ensured by review of data by the Alliance Statistics and Data Management Center and by the study chairperson following Alliance policies. All analyses were based on the study database frozen on 9 November 2009.

## Results

Of the 1264 enrolled patients, 394 case subjects had sufficient tumour tissue for evaluation of *ZNF331* promoter methylation status. Of these 394 case subjects, 9 (2.2%) were removed from the analysis cohort due to assay failure. In general, demographic and clinicopathologic features were similar among the 385 subjects included in the analysis group as compared to the remainder of the trial population (Supplemental Table S2). Median follow-up for DFS analyses among the 385 evaluable subjects was 7.66 years (IQR: 7.58 to 7.76).

### Baseline characteristics according to ZNF331 methylation status

Among the 385 case subjects analysed, *ZNF331* was methylated in 267 (69.35%) specimens, and 118 (30.65%) were unmethylated (unm*ZNF331*). Demographic features of the study population are described in Table 1 (and were noted to be similar). Patients with *mZNF331* were slightly older at diagnosis (median age 63 [IQR 54.0, 70.0] versus 59 years [IQR 51.0, 67.0], *p* = 0.01). There were 187 patients treated with FU/LV (71.7% methylated, 28.3% unmethylated), and 198 patients were treated with IFL (67.2% methylated, 32.8% unmethylated). Otherwise, there was no significant difference between the two groups regarding randomized treatment arm, sex, performance status (PS), or number of positive lymph nodes.

**Table 1.** Demographics of the study population by *ZNF331* promoter methylation status.

	Methylated (*N* = 267)	Unmethylated (*N* = 118)	Total (*N* = 385)	*p*-value^a^
**Age**				0.01
Median (Years)	63.0	59.0	62.0	
Q1, Q3 (Years)	54.0, 70.0	51.0, 67.0	53.0, 69.0	
**Sex**				0.71
Male	148 (55.4%)	63 (53.4%)	211 (54.8%)	
Female	119 (44.6%)	55 (46.6%)	174 (45.2%)	
**Performance Status**				0.53
0	203 (76.6%)	93 (79.5%)	296 (77.5%)	
1–2	62 (23.4%)	24 (20.5%)	86 (22.5%)	
Missing	2	1	3	
**Treatment Arm**				0.34
FU/LV	134 (50.2%)	53 (44.9%)	187 (48.6%)	
IFL	133 (49.8%)	65 (55.1%)	198 (51.4%)	
**Tumor Site**				<0.0001
Distal	173 (65.8%)	46 (39.3%)	219 (57.6%)	
Proximal	90 (34.2%)	71 (60.7%)	161 (42.4%)	
Missing	4	1	5	
**T-Stage**				0.31
T1/T2	21 (8.0%)	15 (12.9%)	36 (9.5%)	
T3	222 (84.1%)	93 (80.2%)	315 (82.9%)	
T4	21 (8.0%)	8 (6.9%)	29 (7.6%)	
Missing	3	2	5	
**Number of Nodes Sampled**				0.28
Median	13.0	12.0	13.0	
Q1, Q3	9.0, 19.0	8.0, 17.0	9.0, 18.0	
Missing	2	1	3	
**Number of Positive Nodes**				0.89
Median	3.0	3.0	3.0	
Q1, Q3	1.0, 5.0	1.0, 5.0	1.0, 5.0	
Missing	2	1	3	
**Extramural Vascular Invasion**				0.58
Absent	233 (90.3%)	105 (92.1%)	338 (90.9%)	
Present	25 (9.7%)	9 (7.9%)	34 (9.1%)	
Missing	9	4	13	
**Perineural Invasion**				0.84
Absent	241 (92.3%)	105 (92.9%)	346 (92.5%)	
Present	20 (7.7%)	8 (7.1%)	28 (7.5%)	
Missing	6	5	11	
**Lymphovascular Invasion**				0.70
Absent	177 (67.6%)	80 (69.6%)	257 (68.2%)	
Present	85 (32.4%)	35 (30.4%)	120 (31.8%)	
Missing	5	3	8	
**Histologic Grade**				0.99
Grade 1/2	201 (76.1%)	89 (76.1%)	290 (76.1%)	
Grade 3/4	63 (23.9%)	28 (23.9%)	91 (23.9%)	
Missing	3	1	4	
**MMR Status**				0.07
pMMR	216 (85.0%)	102 (91.9%)	318 (87.1%)	
dMMR	38 (15.0%)	9 (8.1%)	47 (12.9%)	
Missing	13	7	20	
***BRAF***^**V600E**^				0.003
Wildtype	207 (81.5%)	103 (93.6%)	310 (85.2%)	
Mutant	47 (18.5%)	7 (6.4%)	54 (14.8%)	
Missing	13	8	21	
***KRAS*****mutation**				0.049 [2]
Wildtype	160 (63.0%)	81 (73.6%)	241 (66.2%)	
Mutant	94 (37.0%)	29 (26.4%)	123 (33.8%)	
Missing	13	8	21	
***TP53*****mutation**				0.17
Wildtype	113 (61.1%)	45 (52.3%)	158 (58.3%)	
Mutant	72 (38.9%)	41 (47.7%)	113 (41.7%)	
Missing	82	32	114	
**CIMP Status**				<0.0001
CIMP^–^	184 (69.2%)	113 (97.4%)	297 (77.7%)	
CIMP^+^	82 (30.8%)	3 (2.6%)	85 (22.3%)	
Missing	1	2	3	

^a^*p*-values were calculated by χ^2^ test for all categorical variables, and Kruskal-Wallis test for continuous variables (i.e., Age, Number of Nodes Sampled, and Number of Positive Nodes).

Comparing histopathologic features, m*ZNF331* tumours were observed to be more commonly found in a distal tumour location (65.8% of methylated tumours vs. 39.3% in unmethylated specimens, *p* < 0.0001). Other common histopathologic features (including extramural vascular, perineural and lymphovascular invasion and histologic grade) were similar between colon cancers with and without *ZNF331* promoter hypermethylation.

Evaluating molecular features, *ZNF331* promoter hypermethylation was most significantly associated with CIMP+ status (30.8% of methylated tumours, compared to 2.6% of unmethylated tumours, *p* < 0.0001). Additional significant associations were observed with *BRAF*^V600E^ mutation (18.5% of methylated, compared to 6.4% of unmethylated tumours, *p* = 0.003) and *KRAS* mutations (37.0% of methylated tumours, compared to 26.4% of unmethylated tumours, *p* = 0.049).

### ZNF331 promoter methylation status and patient survival in stage III colon cancer

**Figure 1.**
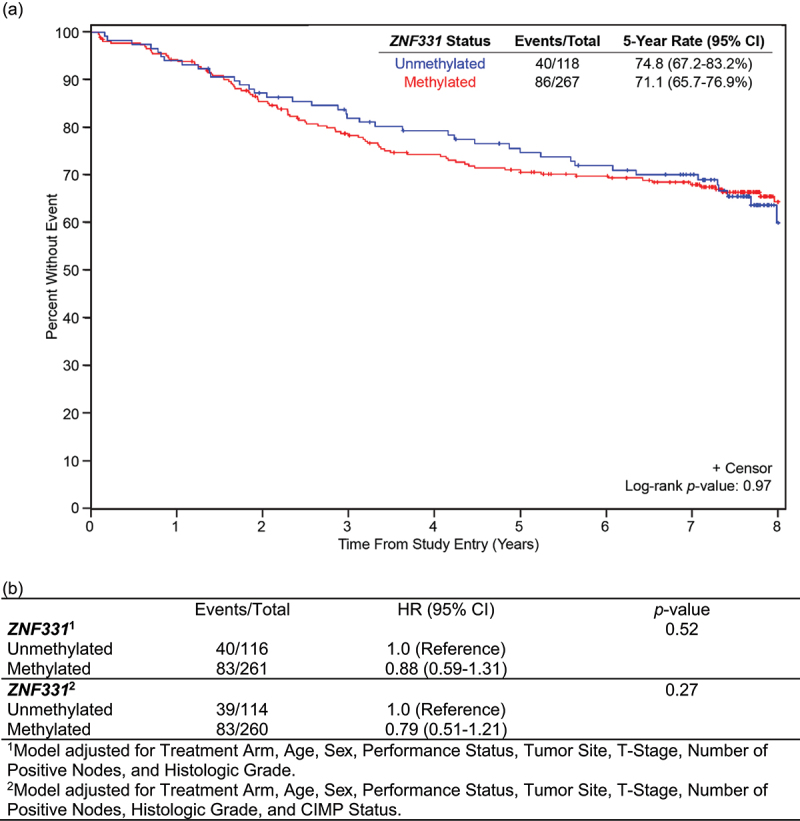
Overall survival by *ZNF331* promoter methylation status. (a) OS is based on *ZNF331* promoter methylation status in the total analysis population (*N* = 385). There was no significant difference in OS in patients with methylated versus unmethylated *ZNF331* colon cancers. (b) Evaluation of *ZNF331* promoter methylation status as a prognostic marker of OS.

A Kaplan-Meier analysis was performed to determine whether *ZNF331* promoter methylation status was associated with survival. There was no observed significant association between *ZNF331* promoter methylation status and OS or DFS. At 5 years, OS was 74.8% (95% CI 67.2–83.2%) for patients with unm*ZNF331* colon cancers compared to 71.1% (95% CI 65.7–76.9%) for patients with m*ZNF331* colon cancers, *p* = 0.97 (Figure 1(a)). In the multivariable Cox regression analysis, there was no observed prognostic association of *ZNF331* promoter methylation status with OS when adjusting for other predictors of patient survival, including age, sex, performance status, tumour site, stage, node status, histologic grade, and CIMP status (*p* = 0.27) (Figure 1(b)).

**Figure 2.**
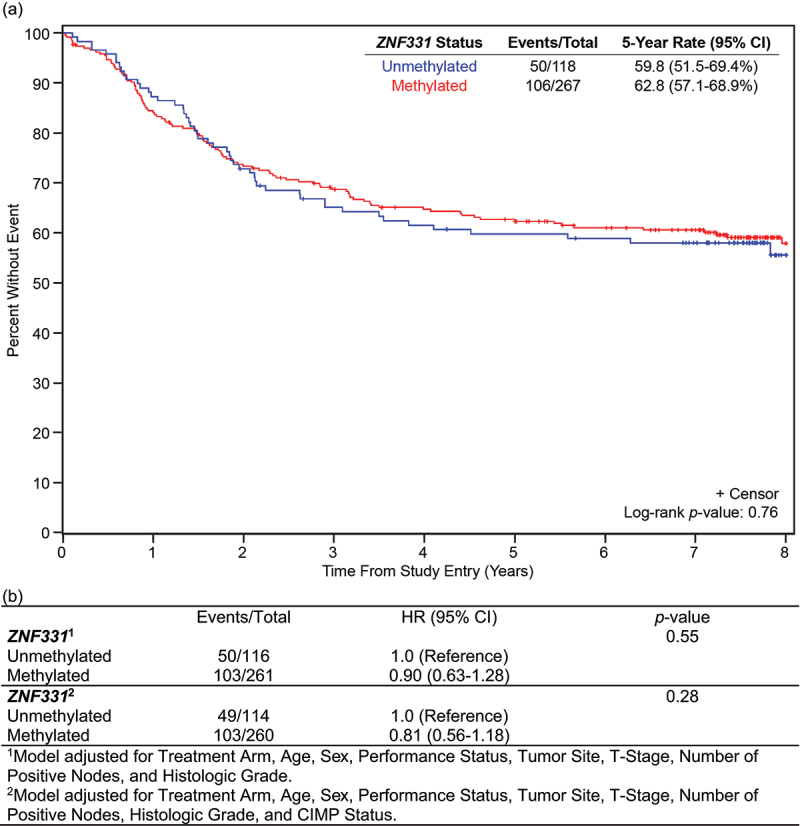
Disease-free survival by *ZNF331* promoter methylation status. (a) DFS OS is based on *ZNF331* promoter methylation status in the total analysis population (*N* = 385). There was no significant difference in DFS in patients with methylated versus unmethylated *ZNF331* colon cancers. (b) Evaluation of *ZNF331* promoter methylation status as a prognostic marker of DFS.

Similarly, at 5 years, DFS was 59.8% (95% CI 51.5–69.4%) for patients with unm*ZNFF331* colon cancers compared to 62.8% (95% CI 57.1–68.9%) with m*ZNF331* colon cancers, *p* = 0.76 (Figure 2(a)). There was no observed prognostic association of *ZNF331* promoter methylation status with DFS, when adjusting for other predictors of patient survival, including age, sex, performance status, tumour site, stage, node status, histologic grade, and CIMP status using multivariable Cox regression analysis (*p* = 0.28) (Figure 2(b)).

### ZNF331 promoter methylation status and standard molecular markers of colon cancer

The interaction between *ZNF331* promoter methylation status and specific prognostic molecular markers of colon cancer was also evaluated. No statistically significant interaction was observed between *ZNF331* promoter methylation status and molecular subgroup based on MMR status (interaction, *p* = 0.43), *BRAF*^V600E^ mutation status (interaction, *p* = 0.64), *KRAS* mutation status (interaction, *p* = 0.21), *TP53* (interaction, *p* = 0.72), CIMP status (interaction, *p* = 0.43) (Supplemental Figure 1). Similarly, no statistically significant association was observed between *ZNF331* promoter methylation status and DFS when accounting for any of these molecular subgroups (Supplemental Figure 2).

### Predictive role of ZNF331 promoter methylation status for irinotecan-based therapy

**Figure 3.**
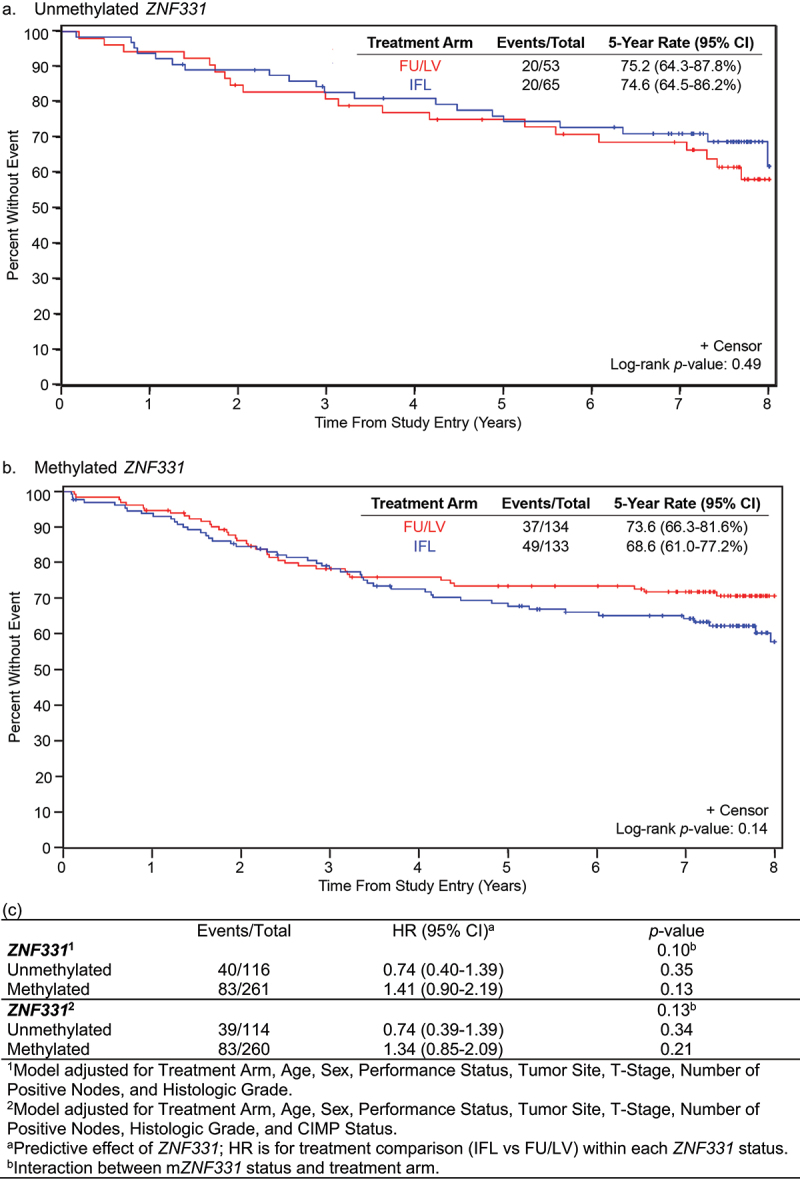
Overall survival (OS) for patients treated with FU/LV vs IFL based on *ZNF331* promoter methylation status. Interaction of *ZNF331* promoter methylation status and treatment arm on disease-free survival: (a) unmethylated *ZNF331* and (b) methylated *ZNF331*. There was no observed difference in OS based on a two-way interaction model between treatment arm and *ZNF331* promoter methylation status. (c) Evaluation of *ZNF331* promoter methylation status as a predictive marker of OS.

**Figure 4.**
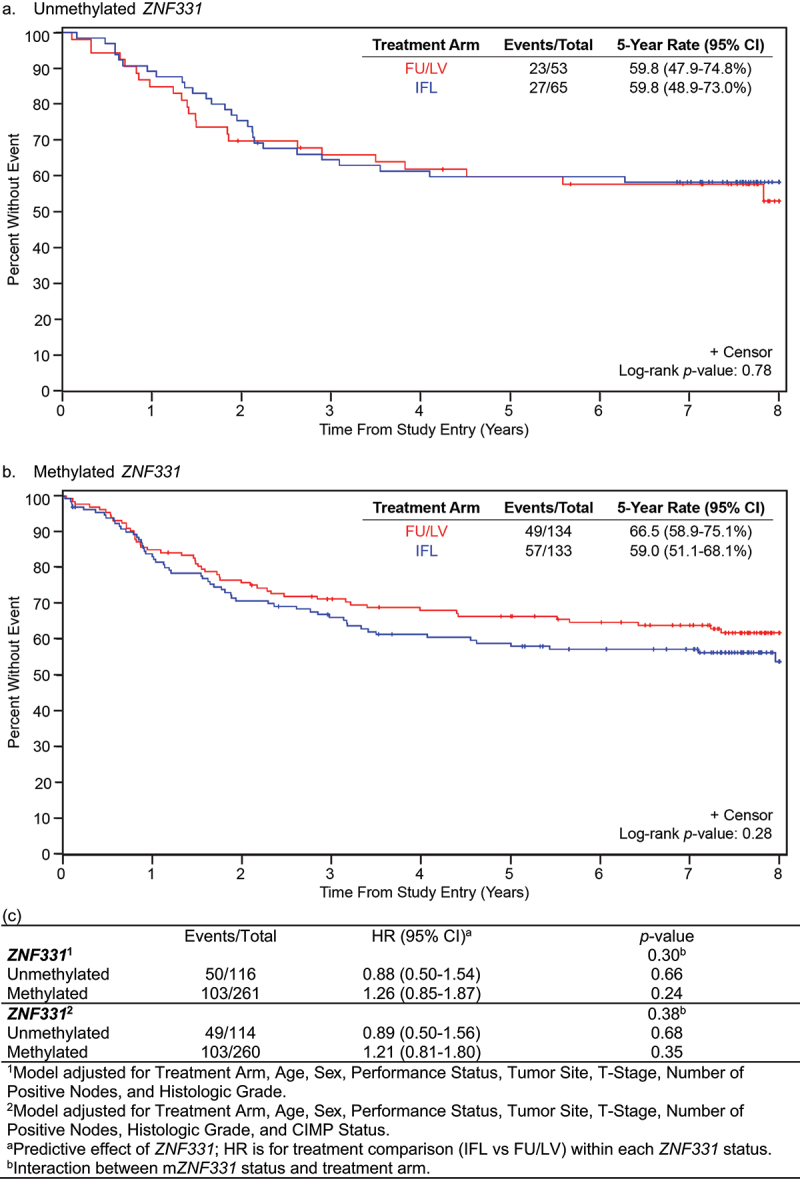
Disease-free survival (DFS) for patients treated with FU/LV vs IFL based on *ZNF331* promoter methylation status. Interaction of *ZNF331* promoter methylation status and treatment arm on disease-free survival: (a) unmethylated *ZNF331* and (b) methylated *ZNF331*. There was no observed difference in DFS based on a two-way interaction model between treatment arm and *ZNF331* promoter methylation status. (c) Evaluation of *ZNF331* promoter methylation status as a predictive marker of DFS.

The interaction between *ZNF331* promoter methylation status and treatment arm was evaluated. In both FU/LV and IFL treatment arms, *ZNF331* promoter methylation status was not significantly associated with OS (Figure 3) or DFS (Figure 4). Five-year OS for patients with unm*ZNF331* colon cancer was 74.6% (95% CI 64.5–86.2%) with IFL versus 75.2% (95% CI 64.3–87.8%) with FU/LV (*p* = 0.49) (Figure 3(a)). In patients with m*ZNF331* tumours, 5-year OS was 68.6% (95% CI 61.0–77.2%) with IFL versus 73.6% (95% CI 66.3–81.6%) with FU/LV (*p* = 0.14) (Figure 3(b)). There was no significant differential treatment effect in OS using a two-way interaction model between treatment arm and *ZNF331* methylation status when adjusted for clinicopathologic variables (interaction, *p* = 0.10) and CIMP status (interaction, *p* = 0.13) (Figure 3(c)).

For patients with unm*ZNF331* colon cancer, 5-year DFS was equivalent at 59.8% (95% CI 58.9–73.0%) with IFL versus 59.8 (95% 47.9–74.8%) with FU/LV (*p* = 0.78) (Figure 4(a)). In patients with m*ZNF331* colon cancer, 5-year DFS was 59.0 (95% CI 51.1–68.1%) with IFL versus 66.5% (95% CI 58.9–75.1) with FU/LV (*p* = 0.28) (Figure 4(b)). There was no significant differential treatment effect in DFS using a two-way interaction model between treatment arm and *ZNF331* methylation status when adjusted for clinicopathologic variables (interaction, *p* = 0.30) and CIMP status (interaction, *p* = 0.38) (Figure 4(c)).

## Discussion

In this exploratory analysis of patients with stage III, resected colon adenocarcinoma treated in a prospective, randomized trial evaluating adjuvant FU/LV versus IFL (CALGB/Alliance 89,803), we evaluated the utility of *ZNF331* promoter methylation as a prognostic and predictive biomarker. We found that *ZNF331* promoter methylation status is not associated with specific clinicopathologic features, nor is it significantly associated with disease-free or overall survival. Furthermore, while patients whose tumours demonstrated *ZNF331* promoter hypermethylation and received IFL adjuvant therapy had a numerically shorter DFS and OS, this did not reach statistical significance.

Epigenomic alterations are recognized as critical to colorectal cancer carcinogenesis, and emerging epigenetic biomarkers such as CIMP have been shown to be useful as prognostic and predictive markers in CRC [39]. While the majority of these epigenetic markers do not have established prognostic or predictive clinical utility, they have great potential to serve this function. Because significant disease heterogeneity contributes to differences in tumour behaviour and responsiveness to therapy [40], identifying more specific epigenetic biomarkers to enhance patient risk stratification and tailor therapies capable of improving survival outcomes and limiting exposure to toxicities associated with chemotherapy are needed [41].

CIMP remains the most robust predictive and prognostic epigenetic biomarker in CRC, with few other such biomarkers identified to date. It remains unknown whether this is a function of the global methylation changes or if it is being driven by key specific epigenetically altered genes. Two recently identified candidates include transcription factor AP-2 epsilon (*TFAP2E*) and caveolae-associated protein 3 (*CAVIN3*), in which promoter hypermethylation of these two genes are associated with resistance to therapy. *TFAP2E* has been associated with resistance of fluorouracil-based doublet chemotherapy regardless of CRC stage or histopathologic type [42]. Promoter hypermethylation of *CAVIN3*, a protein that regulates activity of *BRCA1*, is associated with resistance to oxaliplatin in CRC cell lines *in vitro* and reduces progression-free survival in patients with metastatic CRC [43].

Prior studies demonstrated that *ZNF331* promoter hypermethylation and its associated loss of expression is both a highly sensitive and specific marker of colorectal cancers [19–21]. In our study, the observed frequency of *ZNF331* promoter methylation is similar to that previously reported [19–21]. Although normal matched tissues were not evaluated in this study, this finding supports prior assertions that m*ZNF331* is a common occurrence in colon cancer.

Discordant results regarding the association of m*ZNF331* with demographic, histopathologic, and molecular features of CRC, as well as its utility as a prognostic marker, have previously been published. Wang et al. [20]. observed a significant association between m*ZNF331* and tumour size ≥5 cm, with no association with tumour location and no association between *KRAS* mutations, *BRAF*^V600E^ mutations, or CIMP status. Kaplan-Meier analysis revealed significantly reduced 5-year DFS (*p* = 0.024) and overall survival (*p* = 0.001) in patients whose tumours demonstrated *ZNF331* methylation compared to those with unmethylated tumours. In contrast, Vedeld et al. demonstrated a significant association of *ZNF331* promoter methylation with localization of the primary tumour in the right colon, microsatellite instability, CIMP+, and *BRAF*^*V600E*^ mutation [21]. While they did not observe a statistically significant reduction in OS by Kaplan-Meier analysis, there was a numerical trend towards reduced survival in patients whose tumours demonstrated *ZNF331* methylation.

Our results are consistent with some, but not all, of the findings of these studies. While we observed a significant association between m*ZNF331*, increased age, and *BRAF*^V600E^ and *KRAS* mutation status as previously reported by Vedeld et al. [21], we did not observe a significant difference in DFS or OS between patients whose tumours exhibited *ZNF331* promoter hypermethylation as compared to those that did not. Furthermore, when accounting for interactions between molecular alterations associated with m*ZNF331*, there was no significant difference in DFS or OS.

In addition to differences in sample size, confounding factors that may account for these conflicting results including differences in patient characteristics (patient ethnicity, inclusion of stage I–II and metastatic patients, as well as patients with rectal cancer), differences in use of systemic therapy, and differences in molecular diagnostics (for example, utilization of four versus five gene panels with application of different percentage of methylated reference cut-offs). The latter highlights a limitation of epigenetic analyses in general, where variability in methods used to define epigenetic alterations such as CIMP may lead to heterogeneity in observed findings. Furthermore, while the patients included in this study were drawn from a large randomized clinical trial with good clinical annotation, this was an exploratory analysis that included a relatively small portion of the patients enrolled in the original trial.

While the results of the overall trial showed no difference in DFS and OS in patients with fully resected stage III colon cancer who received FU/LV as compared to those who received IFL, thereby limiting the utility of IFL in the adjuvant setting, the results of this biomarker study do provide some insight into the potential of *m*ZNF331 as a predictive marker. While not statistically significant, there was a numerical reduction in OS and DFS in patients with cancers that demonstrated *ZNF331* methylation who received IFL compared to those who received FU/LV. Interestingly, in the Vedeld study, a substantial proportion of the patients who met criteria for adjuvant chemotherapy were treated in the post-MOSAIC era. Thus, it would also be useful to understand whether some of the differences observed in survival are due to oxaliplatin exposure. These results would suggest that patients with colon cancers with m*ZNF331* may benefit from FU/LV alone in the adjuvant setting.

Furthermore, if m*ZNF331* is indeed associated with reduced response to combination chemotherapy, this would have larger implications for treatment selection for patients with metastatic disease. Future studies with a larger sample size, inclusion of patients with metastatic disease, and analysis within more modern chemotherapy regimens are needed to understand whether m*ZNF331* promoter hypermethylation is predictive of therapeutic response.

Overall, these data suggest that while promoter hypermethylation of *ZNF331* may be important for CRC carcinogenesis and a common diagnostic feature, the utility of m*ZNF331* as a predictive and prognostic marker remains unclear. Our current study evaluating a subset of patients with stage III colon cancer, suggests limited applicability as a prognostic marker in stage III colon cancer. There may be some applicability of *ZNF331* promoter methylation status as a biomarker predictive of chemotherapy response. However, additional, and larger studies are needed to provide clarity into the use of *ZNF331* promoter methylation status as a prognostic and predictive marker.

## Supplementary Material

Supp fig 1-2 & supp tab 1-2.docx

## Data Availability

De-identified patient data may be requested from Alliance for Clinical Trials in Oncology via http://concepts@alliancenctn.org if data are not publicly available. A formal review process includes verifying the availability of data, conducting a review of any existing agreements that may have implications for the project, and ensuring that any transfer is in compliance with the IRB. The investigator will be required to sign a data release form prior to transfer.

## Support

Research reported in this publication was supported by the National Cancer Institute of the National Institutes of Health under Award Numbers U10CA180821 and U10CA180882 (to the Alliance for Clinical Trials in Oncology), U24CA196171 (to the Alliance for NCTN Biorepository and Biospecimen Resource Grant), UG1CA233328, and UG1CA233373; Health P30-CA015704-44 (S.A.C.), NCI R50CA233042 (M.Y.), T32 CA009515 (LS), National Institutes of Health, Grant/Award Number: P30CA15704, U01CA152756, R01CA220004, U01AG077920, U2CCA271902; Listwin Family Foundation; Cottrell Family Fund; R.A.C.E. Charities; Seattle Translational Tumor Research; Rodger C. Haggitt Endowed Chair; W.H. Geiger Family Foundation to WMG.

Clinicaltrials.gov Identifier: NCT00003835
