# Supplementary material for: Evaluating the utility of ZNF331 promoter methylation as a prognostic and predictive marker in stage III colon cancer: results from CALGB 89803 (Alliance)
Source: Epigenetics. 2024 May 8;19(1):2349980. doi: 10.1080/15592294.2024.2349980 (PMC11085945; doi:10.1080/15592294.2024.2349980)
Supplement: Supp fig 1-2 & supp tab 1-2.docx [file KEPI_A_2349980_SM5765.docx]

**Supplemental Figures**

**Supplemental Table 1**. Primer and probe sequences for the Methylight assays for *ZNF331* and *ALUC4*.

**Supplemental Table 2**. Comparison of case subject demographics included in the analysis group versus all other excluded case subjects among all the entire CALGB/Alliance 89803 cohort.

**Supplemental Figure 1**. Interaction of *ZNF331* methylation status and prognostic and predictive colon adenocarcinoma marker status on overall survival.

**Supplemental Figure 2**.

Interaction of *ZNF331* methylation status and prognostic and predictive colon adenocarcinoma marker status on disease-free survival.

**Supplemental Table 1**. Primer and probe sequences for the Methylight assays for *ZNF331* and *ALU-C4*.

| **Assay** | **Primer/Probe** | **Sequence 5′ → 3′** | **Reference** |
| --- | --- | --- | --- |
| m*ZNF331* | Forward | TTAGAGAAGTTTCGACGTAGTTGGAA | 20 |
|  | Reverse | CGACTCCATTTACGCCGTATAAA |  |
|  | Probe | 6FAM-CGTTCGTTAGTGTTTTTTAG-MGB |  |
| *ALU-C4* | Forward | GGTTAGGTATAGTGGTTTATATTTGTAATTTTAGTA | 25 |
|  | Reverse | ATTAACTAAACTAATCTTAAACTCCTAACCTCA |  |
|  | Probe | 6FAM-CCTACCTTAACCTCCC-MGB |  |

**Supplemental Table 2**. Comparison of case subject demographics included in the analysis group versus all other excluded case subjects among all the entire CALGB/Alliance 89803 cohort.

|  | **Analysis Population**  **(*N* = 385)** | **Non-Analysis Population (*N* = 879)** | **Total**  **(*N* = 1264)** | ***p*-value^1^** |
| --- | --- | --- | --- | --- |
| **Age** | | | | 0.26 |
| Median (Years) | 62.0 | 60.0 | 61.0 |  |
| Q1, Q3 (Years) | 53.0, 69.0 | 51.0, 69.0 | 52.0, 69.0 |  |
| **Sex** | | | | 0.73 |
| Male | 211 (54.8%) | 491 (55.9%) | 702 (55.5%) |  |
| Female | 174 (45.2%) | 388 (44.1%) | 562 (44.5%) |  |
| **Performance Status** | | | | 0.14 |
| 0 | 296 (77.5%) | 629 (73.6%) | 925 (74.8%) |  |
| 1-2 | 86 (22.5%) | 226 (26.4%) | 312 (25.2%) |  |
| Missing | 3 | 24 | 27 |  |
| **Treatment Arm** | | | | 0.58 |
| FU/LV | 187 (48.6%) | 442 (50.3%) | 629 (49.8%) |  |
| IFL | 198 (51.4%) | 437 (49.7%) | 635 (50.2%) |  |
| **Tumor Site** | | | | 0.92 |
| Distal | 219 (57.6%) | 496 (57.9%) | 715 (57.8%) |  |
| Proximal | 161 (42.4%) | 360 (42.1%) | 521 (42.2%) |  |
| Missing | 5 | 23 | 28 |  |
| **T-Stage** | | | | 0.049 |
| T1/T2 | 36 (9.5%) | 121 (14.2%) | 157 (12.7%) |  |
| T3 | 315 (82.9%) | 658 (77.1%) | 973 (78.9%) |  |
| T4 | 29 (7.6%) | 74 (8.7%) | 103 (8.4%) |  |
| Missing | 5 | 26 | 31 |  |
| **Number of Nodes Sampled** | | | | 0.55 |
| Median | 13.0 | 12.0 | 12.0 |  |
| Q1, Q3 | 9.0, 18.0 | 8.0, 19.0 | 8.0, 18.0 |  |
| Missing | 3 | 24 | 27 |  |
| **Number of Positive Nodes** | | | | 0.14 |
| Median | 3.0 | 2.0 | 2.0 |  |
| Q1, Q3 | 1.0, 5.0 | 1.0, 4.0 | 1.0, 4.0 |  |
| Missing | 3 | 21 | 24 |  |
| **Extramural Vascular Invasion** | | | | 0.88 |
| Absent | 338 (90.9%) | 760 (91.1%) | 1098 (91.0%) |  |
| Present | 34 (9.1%) | 74 (8.9%) | 108 (9.0%) |  |
| Missing | 13 | 45 | 58 |  |
| **Perineural Invasion** | | | | 0.89 |
| Absent | 346 (92.5%) | 766 (92.3%) | 1112 (92.4%) |  |
| Present | 28 (7.5%) | 64 (7.7%) | 92 (7.6%) |  |
| Missing | 11 | 49 | 60 |  |
| **Lymphovascular Invasion** | | | | 0.33 |
| Absent | 257 (68.2%) | 600 (70.9%) | 857 (70.1%) |  |
| Present | 120 (31.8%) | 246 (29.1%) | 366 (29.9%) |  |
| Missing | 8 | 33 | 41 |  |
| **Histologic Grade** | | | | 0.71 |
| Grade 1/2 | 290 (76.1%) | 643 (75.1%) | 933 (75.4%) |  |
| Grade 3/4 | 91 (23.9%) | 213 (24.9%) | 304 (24.6%) |  |
| Missing | 4 | 23 | 27 |  |
| **MMR Status** | | | | 0.29 |
| pMMR | 318 (87.1%) | 461 (84.6%) | 779 (85.6%) |  |
| dMMR | 47 (12.9%) | 84 (15.4%) | 131 (14.4%) |  |
| Missing | 20 | 334 | 354 |  |
| ***BRAF*^V600E^** | | | | 0.44 |
| Wildtype | 310 (85.2%) | 243 (82.9%) | 553 (84.2%) |  |
| Mutant | 54 (14.8%) | 50 (17.1%) | 104 (15.8%) |  |
| Missing | 21 | 586 | 607 |  |
| ***KRAS* mutation** | | | | 0.58 |
| Wildtype | 241 (66.2%) | 186 (64.1%) | 427 (65.3%) |  |
| Mutant | 123 (33.8%) | 104 (35.9%) | 227 (34.7%) |  |
| Missing | 21 | 589 | 610 |  |
| ***TP53* mutation** | | | | 0.11 |
| Wildtype | 158 (58.3%) | 175 (51.8%) | 333 (54.7%) |  |
| Mutant | 113 (41.7%) | 163 (48.2%) | 276 (45.3%) |  |
| Missing | 114 | 541 | 655 |  |
| **CIMP Status** | | | | 0.34 |
| CIMP^–^ | 297 (77.7%) | 174 (74.4%) | 471 (76.5%) |  |
| CIMP^+^ | 85 (22.3%) | 60 (25.6%) | 145 (23.5%) |  |
| Missing | 3 | 645 | 648 |  |
| ^1^*p*-values were calculated by χ^2^ test for all categorical variables, and Kruskal-Wallis test for continuous variables (i.e., Age, Number of Nodes Sampled, and Number of Positive Nodes). | | | | |

**Supplemental Figure 1**. Interaction of *ZNF331* promoter methylation status with prognostic and predictive colon adenocarcinoma marker status on overall survival.


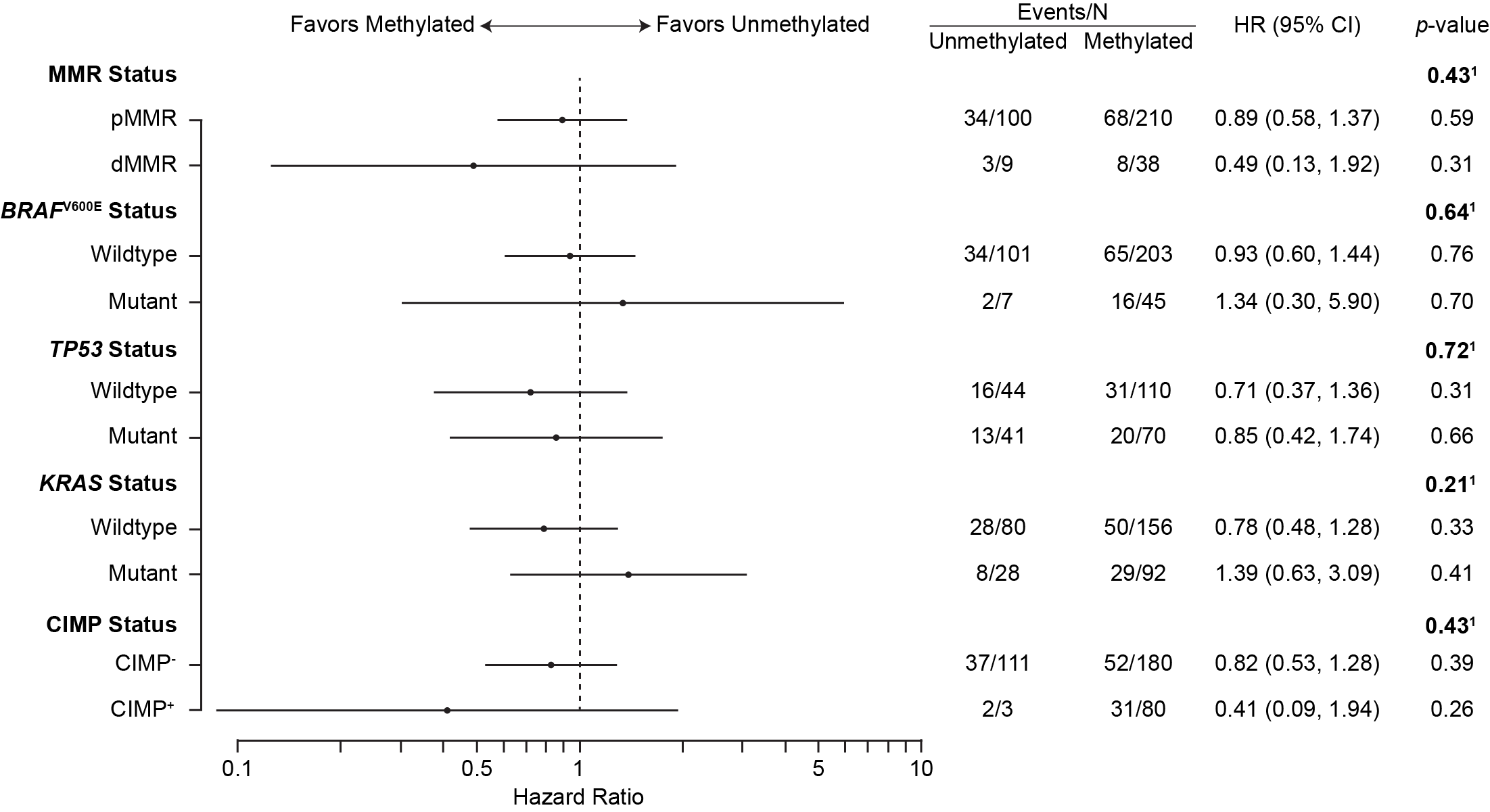


^1^Interaction between m*ZNF331* status and the indicated molecular marker.

Model adjusted for Treatment Arm, Age, Sex, Performance Status, Tumor Site, T-Stage, Number of Positive Nodes, and Histologic Grade.

**Supplemental Figure 2**.

Interaction of *ZNF331* promoter methylation status and prognostic and predictive colon adenocarcinoma marker status on disease-free survival.


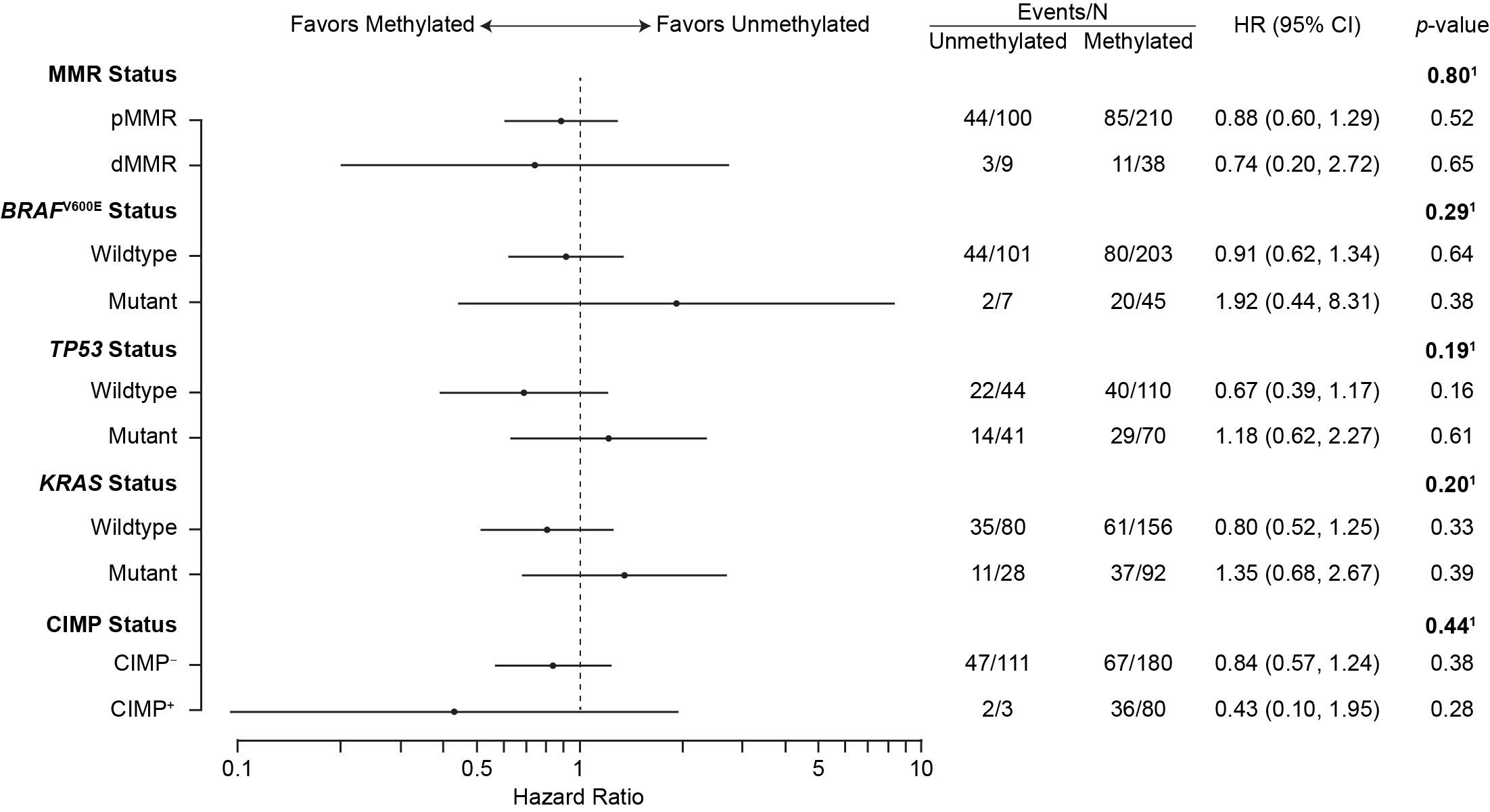


^1^Interaction between m*ZNF331* status and the indicated molecular marker.

Model adjusted for Treatment Arm, Age, Sex, Performance Status, Tumor Site, T-Stage, Number of Positive Nodes, and Histologic Grade.
